# 43 Involvement in active transport is related to organizational factors and physical environment infrastructure

**DOI:** 10.1093/eurpub/ckae114.082

**Published:** 2024-09-26

**Authors:** Kristina Motiejunaite, Brigita Mieziene, Laima Gasiuniene

**Affiliations:** Lithuanian Sports University, Kaunas, Lithuania; Lithuanian Sports University, Kaunas, Lithuania; Lithuanian Sports University, Kaunas, Lithuania

## Abstract

**Purpose:**

Active transport (AT) is considered the most sustainable and accessible mode for travel purposes. AT (walking and cycling) contributes to health-enhancing physical activity as it is a convenient and accessible way to increase physical activity levels. Convenient infrastructure promotion and support are important factors in shift from motor transport towards active travel. The purpose of this study was to determine how organizational factors and neighbourhood physical environment differ between using and not using AT among the Lithuanian young adult population.

**Methods:**

This cross-sectional study included 250 Lithuanian young adults aged 18-39 years, with a mean age of 20.38 years. Of these, 80% were women. AT was measured using the Stages of Change Model-based Questionnaire. Infrastructure was measured by ALPHA Questionnaire–REVISED (Spittals et al., 2010). Organization-related items were developed by study researchers.

**Results:**

Young Lithuanian people preferred cars and/or public transport (62.4%) instead of active transportation (AT) (27.6%) when choosing mobility. The results support a strong link between AT and organizational aspects. AT is more often chosen by people who belong to an organization where environmentally friendly behaviour is encouraged (waste sorting, using AT (p = 0.018); conditions are created to safely store a bicycle or scooter (p = 0.024); that talks about sustainable development, environmentally friendly behaviour, sustainability, etc. (p = 0.034). Quality of the neighbourhood physical environment, such as maintained bicycle paths (p = 0.011), maintained sidewalks (p = 0.049), maintained parks, and other open spaces (p = 0.050) significantly associated with the choice of AT. However, the choice of active transport was not significantly related to the infrastructure of pedestrian zones or paths, infrastructure of special lanes, routes for bicycles, or separation of bicycle paths from car traffic.

**Conclusions:**

Young people’s involvement in AT is influenced by the attitude of the organization in which they work or study towards environmentally friendly behaviour, AT, and sustainable development. The quality of the neighbourhood physical environment also determines the involvement of young people in AT.

